# Identification of ZDHHC17 as a Potential Drug Target for Swine Acute Diarrhea Syndrome Coronavirus Infection

**DOI:** 10.1128/mBio.02342-21

**Published:** 2021-10-26

**Authors:** Yun Luo, Chee Wah Tan, Shi-Zhe Xie, Ying Chen, Yu-Lin Yao, Kai Zhao, Yan Zhu, Qi Wang, Mei-Qin Liu, Xing-Lou Yang, Lin-Fa Wang, Zheng-Li Shi

**Affiliations:** a CAS Key Laboratory of Special Pathogens and Biosafety, Wuhan Institute of Virology, Center for Biosafety Mega-Science, Chinese Academy of Sciences, Wuhan, China; b University of Chinese Academy of Sciences, Beijing, China; c Programme in Emerging Infectious Diseases, Duke-NUS Medical School, Singapore, Singapore; Icahn School of Medicine at Mount Sinai

**Keywords:** CRISPR-Cas9 screen, SADS-CoV, DHHC domain, 2-bromopalmitate

## Abstract

The recent emergence and spread of zoonotic viruses highlights that animal-sourced viruses are the biggest threat to global public health. Swine acute diarrhea syndrome coronavirus (SADS-CoV) is an HKU2-related bat coronavirus that was spilled over from *Rhinolophus* bats to swine, causing large-scale outbreaks of severe diarrhea disease in piglets in China. Unlike other porcine coronaviruses, SADS-CoV possesses broad species tissue tropism, including primary human cells, implying a significant risk of cross-species spillover. To explore host dependency factors for SADS-CoV as therapeutic targets, we employed genome-wide CRISPR knockout library screening in HeLa cells. Consistent with two independent screens, we identified the zinc finger DHHC-type palmitoyltransferase 17 (ZDHHC17 or ZD17) as an important host factor for SADS-CoV infection. Through truncation mutagenesis, we demonstrated that the DHHC domain of ZD17 that is involved in palmitoylation is important for SADS-CoV infection. Mechanistic studies revealed that ZD17 is required for SADS-CoV genomic RNA replication. Treatment of infected cells with the palmitoylation inhibitor 2-bromopalmitate (2-BP) significantly suppressed SADS-CoV infection. Our findings provide insight on SADS-CoV-host interactions and a potential therapeutic application.

## INTRODUCTION

Coronaviruses (CoVs) are enveloped, single-stranded, positive-sense RNA viruses that can infect humans and a wide range of animals with various disease severities, including respiratory, enteric, and neurological pathologies (1). Three notable CoVs, including SARS-CoV in 2003, MERS-CoV in 2012, and SARS-CoV-2 in late 2019, emerged in the past 2 decades and caused severe respiratory disease outbreaks in humans (2). Over the past few decades, pigs, a major livestock species, have suffered from severe CoV diseases with significant economy impacts (3). In pigs, CoVs are typically responsible for severe enteric tract infection and are the second leading cause of diseases after the respiratory tract and nervous system (4). Prior to 2016, at least three different types of porcine CoVs, including transmissible gastroenteritis virus, porcine epidemic diarrhea virus, and porcine deltacoronavirus, have caused severe enteric tract diseases in piglets and were regarded as major threats to swine industry (5).

In 2016 to 2017, a large-scale outbreak of severe fatal diarrhea disease in sucking piglets emerged in Guangdong province, China, and caused significant economic losses in the pig farm industry (6–8). The causative agent, named swine acute diarrhea syndrome coronavirus (SADS-CoV), was rapidly identified to be a novel HKU2-related CoV of bat origin (7). SADS-CoV is highly pathogenic in newborn piglets, with a mortality rate reaching 90% in 5-day-old piglets (7, 9). To our knowledge, SADS-CoV is the first bat-derived CoV that spilled over from bat to infect pig directly in recent years (10). Unlike other CoVs, SADS-CoV species tropism was not restricted to swine but was able to infect cells derived from multiple mammalian species, including primary human cells (11, 12). Currently, no vaccine or antiviral drug against SADS-CoV is available (5).

To date, there are limited numbers of attempts in developing anti-SADS-CoV drugs, mostly targeting virus-directed replication. Li et al. developed an RNA interference (RNAi)-based technology through silencing genomic and subgenomic RNA as a potential therapy against multiple swine enteric CoV infections *in vitro*, including SADS-CoV (13). Edwards et al. demonstrated that the nucleoside analogue GS-5734 (remdesivir) efficiently inhibits SADS-CoV replication *in vitro* (14). However, there has been no report of antiviral strategy targeting host factors for this CoV (15). Genome-scale CRISPR-Cas9 knockout (GeCKO) screening could identify host factors essential for viral replication (16, 17). The CRISPR-Cas9-based screening system has recently been used to identify the essential host factors involved in CoV infections, including SARS-CoV, SARS-CoV-2, MERS-CoV, and multiple seasonal CoVs (18–21).

In the present study, we performed genome-wide CRISPR knockout library screening on SADS-CoV in HeLa cells. We revealed that ZDHHC17 (ZD17) is an important host factor for SADS-CoV infection. Knockout of ZD17 (ZD17^KO^) in HeLa cells strongly decreased SADS-CoV replication. We also demonstrated that 2-BP, an inhibitor of protein palmitoylation, effectively abolishes SADS-CoV infection. This finding provides a potential target for future development of antiviral drugs against the potential zoonotic infection of humans by SADS-CoV.

## RESULTS

### SADS-CoV host dependency factors identified in CRISPR-Cas9 knockout library screen.

**FIG 1:**
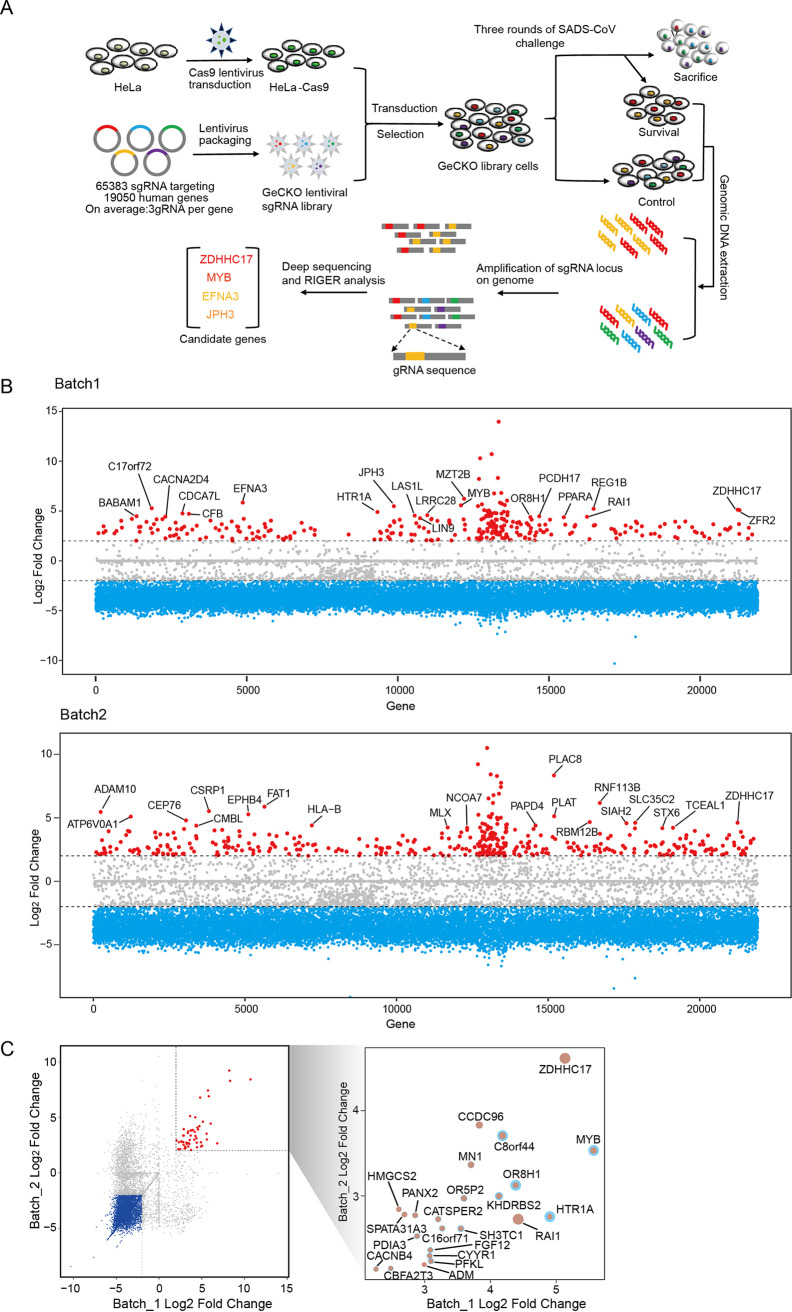
Identification of genes critical for SADS-CoV replication by CRISPR library screening. (A) Genome-wide CRISPR screening strategy. Cas9-expressing HeLa cells are transduced with the genome-wide CRISPR lentivirus and selected with puromycin, followed by SADS-CoV infection. Surviving cells and the mock control are harvested and sgRNA abundance is determined using next-generation sequencing. (B) Bubble plot of data from 2 independent SADS-CoV screens. Red lines denote log_2_ fold change of ±2. (C) Scatterplot comparing log_2_ fold change from 2 independent SADS-CoV screens.

To identify host dependency factors that are necessary for SADS-CoV infection, we performed two independents genome-scale CRISPR knockout screens in HeLa-Cas9 cells (Fig. 1A). To test the quality of the GeCKO plasmid library and cell library, we amplified the single guide RNA (sgRNA) by PCR followed by next-generation sequencing (NGS) and found that 99.9% (65,372/65,383) and 76.6% (50,067/65,383) of the sgRNA were present in the GeCKO plasmid and cell library, respectively (see Fig. S1 in the supplemental material). We then conducted three rounds of SADS-CoV infection on pooled CRISPR-Cas9 knockout cells to enrich SADS-CoV-resistant cells. The genomic DNA (gDNA) of surviving cells were extracted and subjected to NGS to determine the enrichment of the integrated sgRNAs using the MAGeCK suite (22). Our analysis identified 304 and 409 genes that significantly affect SADS-CoV-induced cell death in 2 independent screening (Fig. 1B). Specifically, ZD17 was the top-hit candidate and statistically enriched in 2 independent screens (Fig. 1C) and was selected for further analysis.

10.1128/mBio.02342-21.1 FIG S1 Identification of the GeCKO plasmid and cell library. The sgRNA abundance test of plasmid pools and cell library as well as these genetic screens is shown. Download FIG S1, TIF file, 0.8 MB. Copyright © 2021 Luo et al. 2021 Luo et al. https://creativecommons.org/licenses/by/4.0/ This content is distributed under the terms of the Creative Commons Attribution 4.0 International license.

### SADS-CoV replication was inhibited in ZD17 knockout cells.

**FIG 2:**
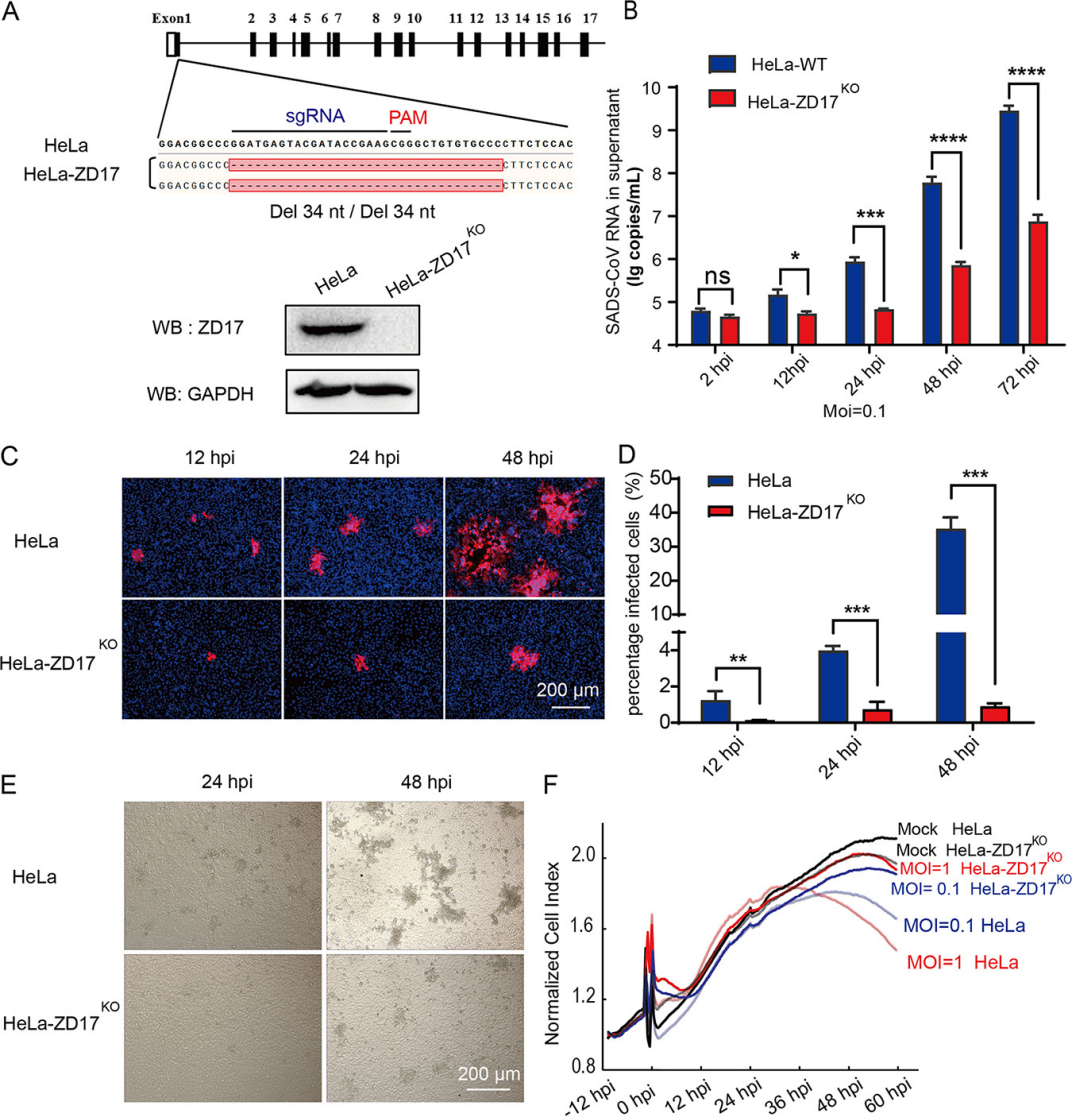
Knockout of ZDHHC17 decreases the SADS-CoV replication. (A) The target sequence in clonal cells is amplified and cloned into the pGEM-T-EASY vector. (Upper) Edited nucleotide sequences in the ZD17 gene alleles are shown according to sequencing analysis. (Lower) The clonal HeLa-ZD17^KO^ and HeLa cells were cultured in 6-well plates, and the expression of endogenous ZD17 was detected by Western blotting with anti-ZD17 rabbit polyclonal antibody. (B) HeLa and HeLa-ZD17^KO^ cells were cultured in 24-well plates and infected with SADS-CoV (MOI, 0.1). At different time points (2, 12, 24, 48, and 72 hpi), RNA was extracted from supernatants and viral genome copies were determined by RT-qPCR with primers targeting the SADS-CoV RdRp gene. (C) Cells from panel B were fixed at 12, 24, and 48 hpi, respectively, and analyzed by IFA using an anti-N protein antibody. (D) The infection rates in panel C were quantified with high content analysis. (E) At 24 h and 48 hpi, CPE was examined to compare the production of infectious progeny virus. (F) The real-time growth and adhesion kinetics of HeLa and HeLa-ZD17^KO^ cells were monitored using a label-free cell-based assay by the xCELLigence real-time cellular analysis (RTCA) system.

To validate the CRISPR hit, we generated ZD17 knockout cells for SADS-CoV replication. Clonal expand ZD17 knockout HeLa (HeLa-ZD17^KO^) cells were confirmed by Sanger sequencing and Western blot analyses (Fig. 2A). Sequencing analysis showed that both alleles of the clonal ZD17 gene had a 34-nucleotide deletion in the first exon of the ZD17, leading to abolishment of protein expression (Fig. 2A). We used real-time quantitative PCR (RT-qPCR) to quantify the viral RNA in supernatants from the SADS-CoV-infected HeLa and HeLa-ZD17^KO^ at different time points postinfection. The results showed that SADS-CoV RNA copies were significantly decreased in the HeLa-ZD17^KO^ cell supernatants after 12 h postinfection (hpi) (Fig. 2B). The expression SADS-CoV nucleocapsid (N) protein was also significantly decreased in HeLa-ZD17^KO^ cells, as revealed by immunofluorescence assay (IFA) (Fig. 2C). Using high-content immune-fluorescence quantification, the percentage of SADS-CoV-infected HeLa cells was 1.25%, 3.99%, and 35.29% at 12, 24, and 48 hpi, respectively, but the infection rate in HeLa-ZD17^KO^ cells was significantly reduced, with percentage of infection of 0.14%, 0.74%, and 0.90% at 12, 24, and 48 hpi, respectively (Fig. 2D). The cytopathic effect induced by SADS-CoV infection in HeLa-ZD17^KO^ cells was decreased compared to that in HeLa cells (Fig. 2E). This finding was further supported by the real-time cell analysis, in which the normalized cell index values of SADS-CoV-infected HeLa cells were decreased at 36 hpi and 48 hpi at a multiplicity of infection (MOI) of 1 and 0.1, respectively. SADS-CoV-infected HeLa-ZD17^KO^ cells did not show significant reduction (Fig. 2F). In addition, the normalized confidence interval (CI) values of HeLa and HeLa-ZD17^KO^ cells were similar prior to virus infection, suggesting that the ZD17 knockout was not cytotoxic.

### ZDHHC17 affects viral genome replication during the SADS-CoV infection cycle.

**FIG 3:**
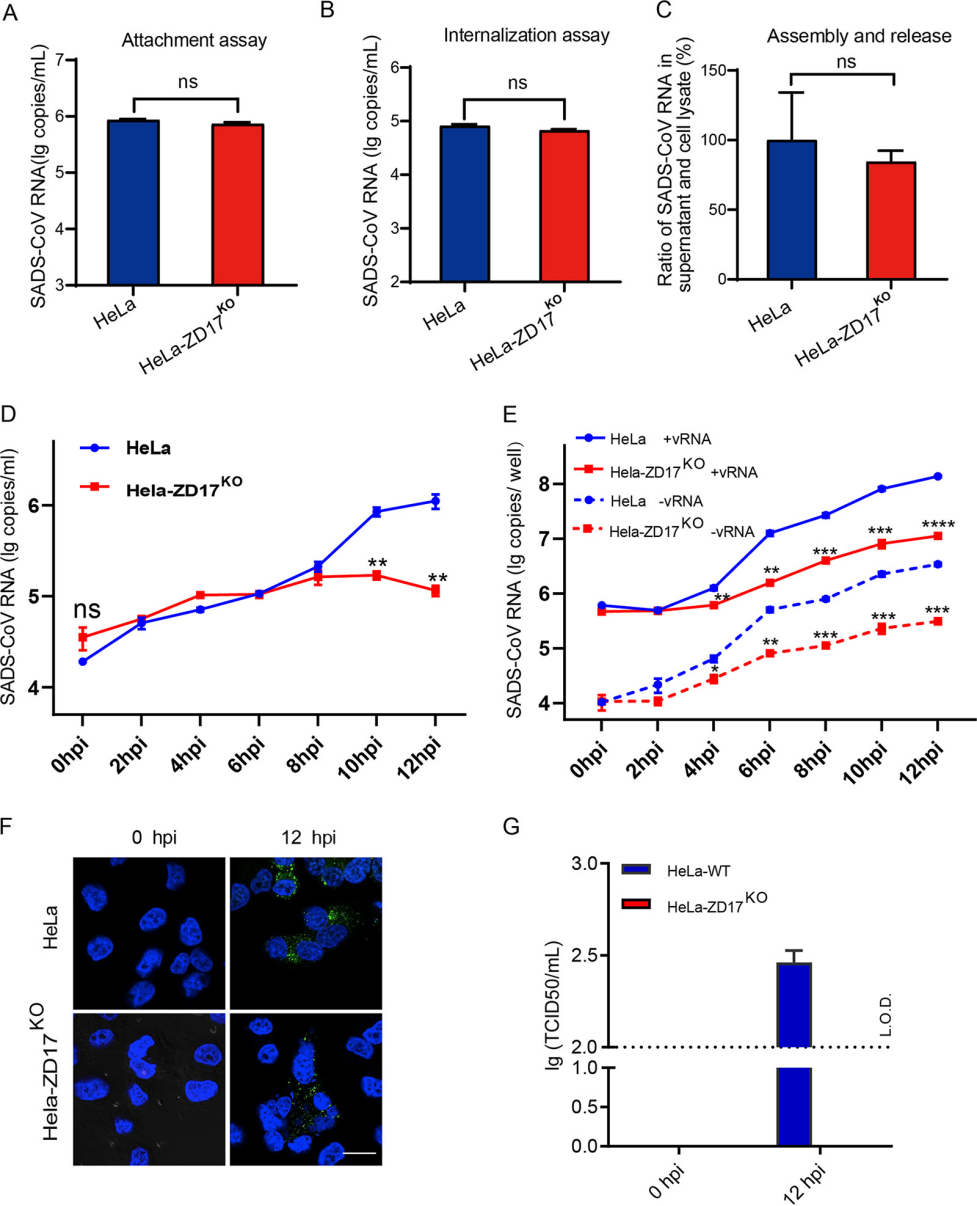
ZDHHC17 is involved in viral RNA synthesis. HeLa and HeLa-ZD17^KO^ cells were inoculated with SADS-CoV (MOI, 0.1) at 4°C for 1 h and washed with cold PBS. (A) The cells were harvested and viral RNA was extracted for determining the virion attachment at the cell surface. (B) The infected cells as described above were further cultured at 37°C for another 1 h. Cells were then harvested after pronase treatment and viral RNA was extracted for determining the virion internalization. (C) At 24 hpi, the ratio of SADS-CoV RNA copy number in the supernatants versus the cell lysates were separately determined by RT-qPCR for assembly and release assay. (D) Quantification of extracellular genomic RNA. (E) Quantification of intracellular positive- and negative-strand RNA at 0, 2, 4, 6, 8, 10, and 12 hpi. (F) Viral RNA was assessed by staining cells with anti-dsRNA antibody followed by confocal microscopy analysis. (G) The infectious virions secreted from HeLa and HeLa-ZD17^KO^ cells were determined by 50% tissue culture infectious dose assays in Vero cells.

To explore the role of ZD17 in the SADS-CoV infection cycle, we examined the effect of ZD17 knockout on viral attachment, internalization, morphogenesis, and RNA replication. No difference was observed in viral RNA level in HeLa and HeLa-ZD17^KO^ cells after incubation of SADS-CoV at 4°C or a 4°C incubation followed by 37°C incubation and pronase treatment, implying ZD17 has no role in viral attachment and entry (Fig. 3A and B). To investigate the role of ZD17 in viral assembly and/or release, HeLa and HeLa-ZD17^KO^ cells were infected with SADS-CoV at an MOI of 0.1. At 24 hpi, the viral RNA level in the infected cells and supernatants were quantified by RT-qPCR, and the ratio of SADS-CoV RNA between cells and supernatant was determined. As shown in Fig. 3C, no significant difference was observed between supernatants and cell lysates, suggesting that ZD17 does not modulate SADS-CoV morphogenesis and release.

To determine whether ZD17 could affect SADS-CoV viral genome replication, extracellular and intracellular viral RNA was monitored in real time at the early stage of infection. First, the extracellular viral genomic RNA (gRNA) level was monitored to estimate the time required for viral replication. The extracellular gRNA level remained stable in HeLa and HeLa-ZD17^KO^ from 0 to 8 hpi but started to increase from 8 hpi, suggesting that one replication cycle of SADS-CoV in HeLa cells took approximately 8 h (Fig. 3D). We used strand-specific RT-qPCR to distinguish the production of positive-strand and negative-strand viral RNA (+vRNA and −vRNA). Intracellular +vRNA and −vRNA copies in HeLa and HeLa-ZD17^KO^ cells showed similar levels at 0 and 2 hpi, followed by a sharp increase at 4 hpi in HeLa cells, while the production of +vRNA and −vRNA was significantly inhibited in HeLa-ZD17^KO^ cells (Fig. 3E and F), which was consistent with the result that almost undetectable virus was produced in HeLa-ZD17^KO^ cells (Fig. 3G). Our data suggested that ZD17 is an important host factor for SADS-CoV replication.

### The DHHC domain of ZD17 is essential for SADS-CoV replication.

**FIG 4:**
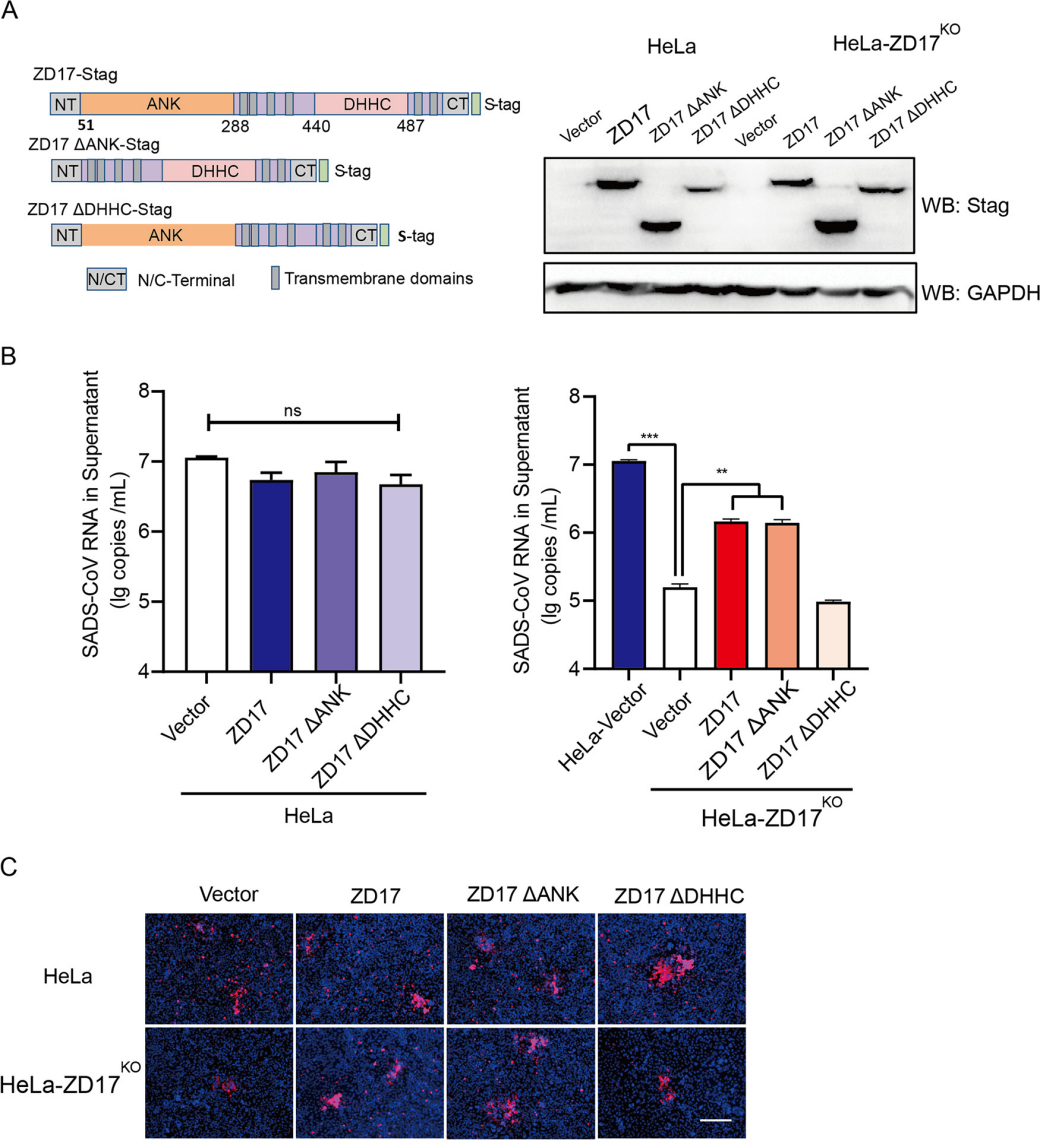
DHHC domain of ZD17 affects SADS-CoV replication. (A) Overexpression of ZD17 and truncation mutants in HeLa and HeLa-ZD17^KO^ cells detected using anti-S-tag mouse monoclonal antibody and HRP-conjugated goat-anti-mouse IgG. (B) HeLa and HeLa-ZD17^KO^ cells were transfected as described for panel A and infected with SADS-CoV (MOI, 0.1). At 48 hpi, RNA was extracted from supernatants, and viral RNA was quantified by RT-qPCR. (C) Cells in panel B were fixed at 48 hpi and analyzed by IFA using an anti-N protein antibody. Scale bar, 100 μm.

Unlike other ZDHHC protein family members, ZD17 contains a conserved DHHC cysteine-rich domain that is required for the palmitoylation activity and also unique ankyrin repeat motifs (ANK) (23). ANK is a scaffold involved in S-acylation substrate recruitment and/or S-acylation-independent functions (24). To determine the functional domain of ZD17 that is involved in SADS-CoV infection, we constructed two ZD17 truncation mutants lacking the ANK domain (ZD17ΔANK) or the DHHC cysteine-rich domain (ZD17ΔDHHC) and full-length ZD17 (Fig. 4A). The overexpressed ZD17ΔANK and ZD17ΔDHHC mutants and full-length ZD17 were determined by Western blotting in HeLa and HeLa-ZD17KO cell lines (Fig. 4A). Viral infection was determined by extracellular viral RNA and intracellular N protein expression. The overexpression of ZD17, ZD17ΔANK, and ZD17ΔDHHC has no significant impact on SADS-CoV replication in HeLa cells, suggesting that endogenous ZD17 expression is sufficient to support SADS-CoV replication (Fig. 4B). Unlike ZD17- and ZD17ΔANK-transfected HeLa-ZD17^KO^ cells in which virus replication was restored to approximately 50% upon infection, virus rescue of ZD17ΔDHHC-transfected HeLa-ZD17^KO^ cells was not successful (Fig. 4B). The subcellular localization of a protein is often associated with its function. We further determined subcellular localization of ZD17 and its truncation mutants in HeLa with and without SADS-CoV infection. We found that ZD17 and its truncation mutants were diffused in the cytoplasm. Infection of SADS-CoV did not affect subcellular localization of ZD17 (Fig. S2). These results demonstrated that the palmitoylation activity of ZD17 in the cytoplasm supports SADS-CoV replication.

10.1128/mBio.02342-21.2 FIG S2 Subcellular localization of overexpressed ZD17 and truncation mutants in HeLa. Cells were transfected with the indicated expression vector (ZD17-FlAG, ZD17ΔANK-FLAG, and ZD17ΔDHHC-FLAG) for 24 h and then were infected with or without SADS-CoV (MOI, 5) for 12 h. Cells were stained with anti-FLAG antibody. Download FIG S2, TIF file, 3.0 MB. Copyright © 2021 Luo et al. 2021 Luo et al. https://creativecommons.org/licenses/by/4.0/ This content is distributed under the terms of the Creative Commons Attribution 4.0 International license.

### 2-BP inhibits SADS-CoV replication.

**FIG 5:**
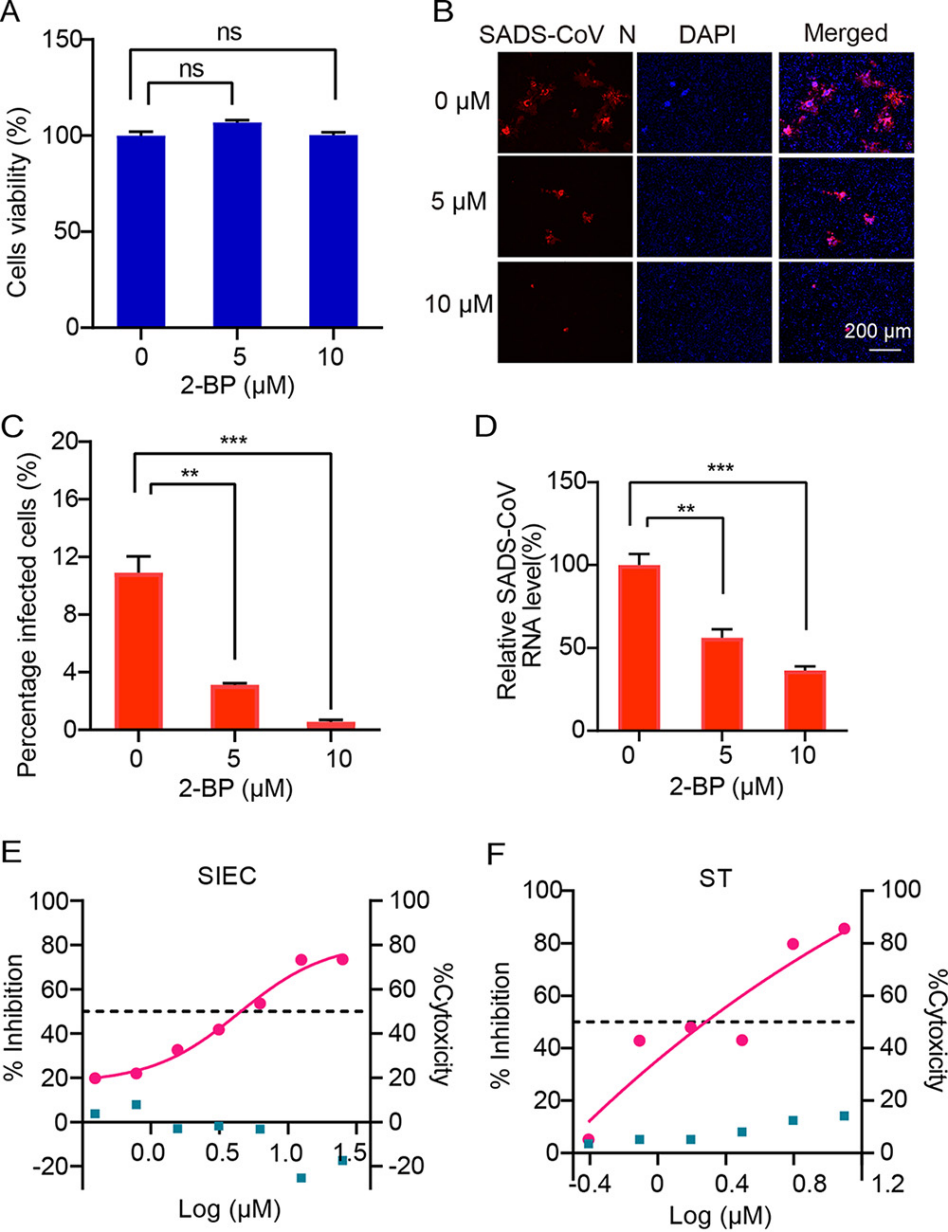
Effect of 2-BP treatment on SADS-CoV infection. (A) Cytotoxicity examination of 2-BP by CCK-8 assay. HeLa cells cultured in the 96-well plates were incubated with 2-BP at the indicated concentrations. At 24 h after incubation, the inhibitor was removed, and CCK-8 reagents (10 μl) were added. After another 2 h of incubation, the optical density at 450 nm was determined. (B) Expression levels of N protein at 24 hpi after treatment with 2-BP. Cells were infected with SADS-CoV at an MOI of 0.2. After 1 h of absorption, the inoculum was removed and cells were maintained in serum-free medium with 2BP. (C) Infection rates in panel B were quantified with high-content analysis. (D) At 24 hpi, RNA was extracted from supernatants and viral RNA was quantified by RT-qPCR. (E and F) Impact of 2-BP treatment on SADS-CoV infection in SIEC and ST cells.

Accumulating evidence indicates that the DHHC cysteine-rich domain is the catalytic center of the palmitoyltransferase (25). A broad-spectrum palmitoylation inhibitor, 2-bromopalmitate (2-BP), was used to determine the role of palmitoylation in SADS-CoV infection. Cell viability assay (CCK-8 assay) showed no cytotoxicity when HeLa cells were treated with 2-BP up to 10 μM (Fig. 5A). The infectivity of SADS-CoV in 2-BP-treated HeLa cells, as determined by IFA, was significantly reduced in a dose-dependent manner (Fig. 5B and C). The viral RNA in the supernatant was also reduced up to 43.76% and 63.57% in 5 μM and 10 μM 2-BP-treated HeLa cells, respectively (Fig. 5D). 2-BP also efficiently inhibited SADS-CoV replication in two swine cell lines (SIEC and ST) (Fig. 5E and F). Altogether, these data supported that the palmitoyltransferase activity of ZD17 is important for SADS-CoV replication.

## DISCUSSION

The ongoing coronavirus disease 2019 (COVID-19) pandemic has claimed over 184 million cases and 3.9 million deaths worldwide (https://covid19.who.int/) as of July 2021. SADS-CoV, a CoV of bat origin, demonstrates wider ranges of species tropism *in vitro* than any other known CoVs, including SARS-CoV, MERS-CoV, and SARS-CoV-2, implying potential risk of spillover from swine or bats to other mammalian hosts, including human (11, 12, 14). Since August 2016, multiple spillover events of SADS-CoV have been reported in south China, which resulted in significant economic losses in the swine industry (7, 26–28). To date, there is no evidence that human can be infected by SADS-CoV (7). However, the possibility cannot be excluded, as SADS-CoV shows broad species tropism and replicates efficiently in primary human lung and intestinal cells (14). Given the three known zoonotic CoV spillover events in recent history, any highly pathogenic CoV should not be neglected, including those in domestic animals. Thus, development of effective vaccines and therapeutics should be prioritized as part of pandemic preparedness in humans as well as for the economically important domestic livestock.

In this study, we performed genome-wide screens for SADS-CoV in human cells to identify druggable host factors as a preemptive approach for development of countermeasures to fight potential human infection. We demonstrated that ZD17 knockout significantly decreased SADS-CoV replication by abolishing viral genome synthesis. Mechanistic studies revealed that palmitoylation activity of the DHHC cysteine-rich domain supports SADS-CoV replication, and palmitoylation inhibitor 2-BP significantly reduces SADS-CoV replication.

ZD17 belongs to a superfamily of palmitoyl-acyltransferases (PATs) catalyzing the palmitate attachment to other protein substrates. ZD17 is highly conserved in mammalian species and shows broad distribution as well as high expression in diverse cells (see Fig. S3 in the supplemental material) (29). The overexpression of ZD17 in HeLa cells does not enhance SADS-CoV infection, suggesting that the constitutive expression of ZD17 could be sufficient in supporting SADS-CoV infection (Fig. 4). ZD17 contains ANK domains involved in protein-protein interactions in addition to the highly conserved DHHC cysteine-rich domains known to account for the palmitoylation activity (24). Apart from PATs, ZD17 also regulates neuronal signal transmission and axonal transport by promoting TrkA–tubulin interaction and may activate the c-Jun N-terminal kinase pathway (30, 31). The multifunctional role of ZD17 is largely attributed to the ANK domain that interacts with the vast diversity of protein substrates (24). The ANK domain serves as a substrate-recruiting module for palmitoylation. Furthermore, it can participate in palmitoylation-independent functions, such as interaction with MAP2K4 to activate JNK/p38 and regulate malignant glioblastoma multiforme development and progression (32). Overexpression of ZD17ΔANK alone was sufficient to rescue SADS-CoV replication in ZD17 knockout cells, suggesting the ANK domain does not contribute to SADS-CoV replication (Fig. 4).

10.1128/mBio.02342-21.3 FIG S3 ZD17 is highly conserved in mammalian species and shows broad distribution as well as constitutive expression in diverse cells. (A) Sequence alignment of ZD17 from human, pig, mouse, pangolin, and four bat species. The highly conserved ANK and DHHC domain are boxed in black and red, respectively. (B) The protein expression data were obtained from the human protein atlas. Download FIG S3, TIF file, 2.5 MB. Copyright © 2021 Luo et al. 2021 Luo et al. https://creativecommons.org/licenses/by/4.0/ This content is distributed under the terms of the Creative Commons Attribution 4.0 International license.

Palmitoylation is a posttranslation modification involved in the addition of palmitic acid to the cysteine residues, which affects the function of proteins by regulating their transport, stability, and membrane localization (33). Several studies suggested that the palmitoylation on the CoV envelope (E) and spike (S) proteins could enhance virion production (34). The palmitoylations of mouse hepatitis virus (MHV) E and S proteins are essential for virion assembly, and 2-BP treatment reduced palmitoylation of the mouse hepatitis virus spike protein and its infectivity *in vitro* (35–37). The palmitoylations of cysteine-rich domains of SARS-CoV S glycoprotein were found to be important for spike-mediated cell fusion (38). On the contrary, another study revealed that the palmitoylation of SARS-CoV E did not affect virus-like particle production *in vitro*, suggesting that the palmitoylated E is not required for SARS-CoV assembly (39). To date, palmitoylation of viral proteins has been associated with the entry of enveloped viruses into target cells by spike-mediated membrane fusion and assembly/release of virus particles from infected cells (40). In our study, we demonstrated that ZD17 is not involved in SADS-CoV entry, assembly, and release (Fig. 3) but is involved in genome synthesis. ZD family proteins, including ZD2 and ZD19, have been shown to facilitate Chikungunya virus (CHIKV) replication by interacting and palmitoylating cysteines at sites 417 to 419 of CHIKV nonstructural protein (nsp1) (41). Previous studies demonstrated that the nsp1 palmitoylations of *Alphavirus*, including CHIKV, Semliki Forest virus, and Sindbis virus, are important for the cellular membrane localization and natural function (41–43). For coronavirus, ORF1ab generate 16 nonstructural proteins that are involved in genome replication. Among them, nsp3, nsp4, and nsp6 were known to be membrane bound (44). We found that only SADS-CoV nsp3 had three potential key residues for palmitoylation based on our predictions (Table S1). Considering that 2-BP, a palmitoylation inhibitor, could significantly reduce SADS-CoV replication, it is worth investigating whether ZD17 facilitates SADS-CoV replication via palmitoylating nsp3. To our knowledge, we are the first to describe the role of palmitoylation in CoV genome replication.

10.1128/mBio.02342-21.4 TABLE S1 Palmitoylation sites of nsp3, nsp4, and nsp6. Protein palmitoylation sites of nsp3, nsp4, and nsp6 were predicted via http://csspalm.biocuckoo.org/. The potential palmitoylation sites were highlighted in red. Download Table S1, DOCX file, 0.01 MB. Copyright © 2021 Luo et al. 2021 Luo et al. https://creativecommons.org/licenses/by/4.0/ This content is distributed under the terms of the Creative Commons Attribution 4.0 International license.

In conclusion, we demonstrated that ZD17 could be a host-directed therapy target against SADS-CoV replication. Currently, no specific ZD17 inhibitor is commercially available. We have demonstrated the antiviral potential of a broad-spectrum palmitoyltransferase inhibitor, 2-BP. However, the use of a broad palmitoyltransferase inhibitor may lead to unwanted side effects. Therefore, it is necessary to develop ZD17-specific inhibitor(s) as a host-directed therapy target for SADS-CoV.

## MATERIALS AND METHODS

### Cell and virus.

HeLa and HEK293T cells were maintained in Dulbecco’s modified Eagle’s medium (DMEM; Gibco) supplemented with 10% fetal bovine serum (FBS; Gibco). SADS-CoV isolate CN/GDWT/2017 (GenBank accession number MG557844) was propagated and titrated on Vero cells in DMEM supplemented with 8 μg/ml trypsin. HeLa and gene-edited cells were inoculated with SADS-CoV and then maintained in serum-free medium with 2% tryptose phosphate broth.

### Plasmids and reagents.

GeCKOv2 human libraries with 2-vector format (lentiGudie-Puro and LentiCas9-BLAST) were obtained from a commercial source (1000000049; Addgene). The cytomegalovirus (CMV) promoter-driven expression vector, pCAGGS, was used to overexpress ZD17 and two ZD17 truncation mutants, and each gene fused with an S-tag (KETAAAKFERQHMDS) or FLAG at the C terminus. The following antibodies were used in this study: ZDHHC17 polyclonal antibody (15465-1-AP; Proteintech), DYKDDDK-Tag(3B9) mouse antibody (M20008; Abmart), anti-dsRNA MAb J2 (J2-1702; Scicons, Hungary), Cy3-conjugated anti-rabbit IgG (ab6939; Abcam), and DyLight488 anti-mouse IgG (ab96879; Abcam). Anti-S-tag mouse monoclonal antibody was made in-house.

### Lentivirus production and titer.

The GeCKOv2 human libraries were amplified in Stellar electrocompetent cells (TakaRa, Japan), and the quality of the amplified sgRNA library was determined by next-generation sequencing. HEK293T cells were seeded into 10-cm culture dishes, grown to 80% confluence, and then cotransfected with the pooled sgRNA library and lentiviral helper plasmids (pMD2.G, pMDLg/PRRE, and pRSV/ReV). At 48 h posttransfection, the lentivirus-containing supernatant was harvested into a tube. Cell debris was removed by centrifugation at 2,000 × *g* for 5 min, and the supernatant was clarified with a 0.45-μm filter. Lentiviral titer was determined in HeLa-Cas9 with replacing fresh growth medium containing 2 μg/ml puromycin every 3 to 4 days after transduction.

### Generation of GeCKO library cells and SADS-CoV infection.

We generated a HeLa cell clone stably expressing Cas9 components (HeLa-Cas9). Next, we transduced these cells with a lentiviral gRNA library targeting 19,050 genes (3 sgRNAs/gene) at an MOI of 0.3 to ensure that most cells received only one viral construct. After 72 h, the transduced HeLa-Cas9 cells were selected with 2 μg/ml puromycin for 7 days. After that, the cells were infected with SADS-CoV at an MOI of 0.1 and washed with PBS at 72 hpi to remove dead cells, and then fresh medium was added to surviving clones. Surviving cells were cultured and used for subsequent SADS-CoV infection rounds. The cells were resistant to this viral infection after three rounds of infections. At the end of the screen, we harvested cells for genome extraction and amplified the sgRNA for next-generation sequencing.

### Generation of the ZDHHC17-KO cell lines.

The sgRNA (5′-GGATGAGTACGATACCGAAG-3′) targeting the ZD17 gene was inserted into the backbone plasmid of lentiCRISPR-v2 vector (Addgene) at the BsmBI site. Constructs were cotransfected with lentiviral helper plasmids mentioned above using Lipofectamine 3000 into HEK293T cells. The lentiviruses produced above were used to transduce HeLa cells, followed by puromycin selection at 2 μg/ml for 1 week. Surviving cells were diluted into 96-well plates for clonal expansion. The ZD17 gene-edited cell lines (ZD17^KO^) were validated by DNA sequencing and Western blotting.

### Western blotting.

Cells were cultured in 24-well plates, harvested with radioimmunoprecipitation assay (RIPA) lysis buffer, and boiled for 10 min with sample loading buffer. The protein samples were separated by SDS-PAGE and transferred to a polyvinylidene difluoride (PVDF) membrane. The PVDF membranes were blocked with 5% bovine serum albumin and probed with appropriate primary and secondary antibodies. Proteins were detected using a Western blot analysis system (Bio-Rad).

### RTCA.

The real-time growth and adhesion kinetics of HeLa cells were monitored using a label-free cell-based assay by the xCELLigence real-time cell assay (RTCA) system (RTCA DP; ACEA Biosciences, San Diego, CA, USA). Briefly, 100 μl of DMEM supplemented with 10% FBS was placed in each well of an E-plate 16 (gold-microelectrode array integrated E-plate). A total of 2 × 10^4^ cells (HeLa and HeLa-ZD17^KO^) were seed into the wells containing 100 μl of culture medium. Cell index values, which reflected the number of adherent cells, were measured by continuous impedance recordings every 15 min. After overnight incubation, cells were infected with SADS-CoV suspension (MOI of 1 and 0.1). E-Plate 16 was then incubated in the RTCA SP Station, and the cell index values were recorded every 15 min. The cell growth index was normalized at each time point.

### Cell viability assay.

Cell viability was assessed using CCK-8 assays according to the manufacturer’s instructions (MedChem Express, USA). Cells were seeded into 96-well plates containing 100 μl/well of cell culture medium and incubated in a 37°C incubator overnight. The culture medium was removed, and fresh medium containing the compounds at different concentrations was added. Next, 10 μl of the cell viability reagent was directly added to each well, followed by incubation for 2 h at 37°C, protected from direct light with tin foil. The absorbance values at 450 nm were measured to determine cell viability.

### Virion attachment and internalization assay.

HeLa and HeLa-ZD17^KO^ cells were seed in 24-well plates in advance. Precooled cells were infected at an MOI of 1 for 1 h on ice. The supernatant was then removed, and the cells were washed 3 times with ice-cold PBS to remove unbound virus particles. Cells were lysed with TRIzol reagent (Invitrogen), and viral RNA was extracted for determining the virion attachment at the cell surface. To determine internalization, the infected cells as described above were further cultured at 37°C for another 1 h. The cells were treated with 1 mg/ml of pronase in PBS to remove the bound but noninternalized virus particles. After the final wash, the cells were harvested for RNA extraction.

### Quantitative RT-PCR.

Extracellular viral RNA was extracted from supernatants of SADS-CoV-inoculated cells at different time points using the High Pure viral RNA kit (Roche, Basel, Switzerland). Intracellular viral RNA was extracted from cell lysate using a magLEAD nucleic acid purification system (Precision System Science Co., Ltd., Japan). Specific primers for the *RdRp* gene of SADS-CoV (forward, 5′-GCGATGAGATGGTCACTAAAGG-3′; reverse, 5′-GGAATACCCATACCTGGCATAAC-3′) were designed according to the reference sequence (GenBank accession number MF094681). For SADS-CoV strand-specific real-time RT-PCR, *RdRp*-forward primer and *RdRp*-reverse primer were used to synthesize viral minus-strand and plus-strand RNA, respectively. A real-time one-step quantitative RT-PCR assay was used to determine the SADS-CoV genomic RNA using the HiScript II one-step qRT-PCR SYBR green kit (Vazyme, China) as described previously (12).

### Immunofluorescence assay.

Cell susceptibility was determined by immunofluorescence assay (IFA) targeting N protein. Briefly, cells were washed after SADS-CoV infection, fixed with 4% paraformaldehyde for 30 min at room temperature, and then treated with 0.1% Triton X-100. The fixed cells were incubated with rabbit polyclonal antibody against the SADS-CoV N protein followed by incubation with Cy3-conjugated anti-rabbit IgG (ProteinTech, China) and then by 0.01% 4′,6-diamidino2-phenylindole (DAPI) staining for 15 min to detect nuclei. After three washes with PBS, cells were observed under a fluorescence microscope.

### Statistics.

Statistical significance was assessed using a paired Student's *t* test in GraphPad Prism 8 (GraphPad Software Inc., La Jolla, CA). Data represent the averages from at least triplicate standard errors of the means (SEM), unless stated otherwise. In figures, significant differences are represented as asterisks: *, *P* < 0.05; **, *P* < 0.01; ***, *P* < 0.001; ****, *P* < 0.0001; ns, no significant difference.
