# Supplementary figures and images for: Identification of ZDHHC17 as a Potential Drug Target for Swine Acute Diarrhea Syndrome Coronavirus Infection
Source: mBio. 2021 Oct 26;12(5):e02342-21. doi: 10.1128/mBio.02342-21 (PMC8546599; doi:10.1128/mBio.02342-21)

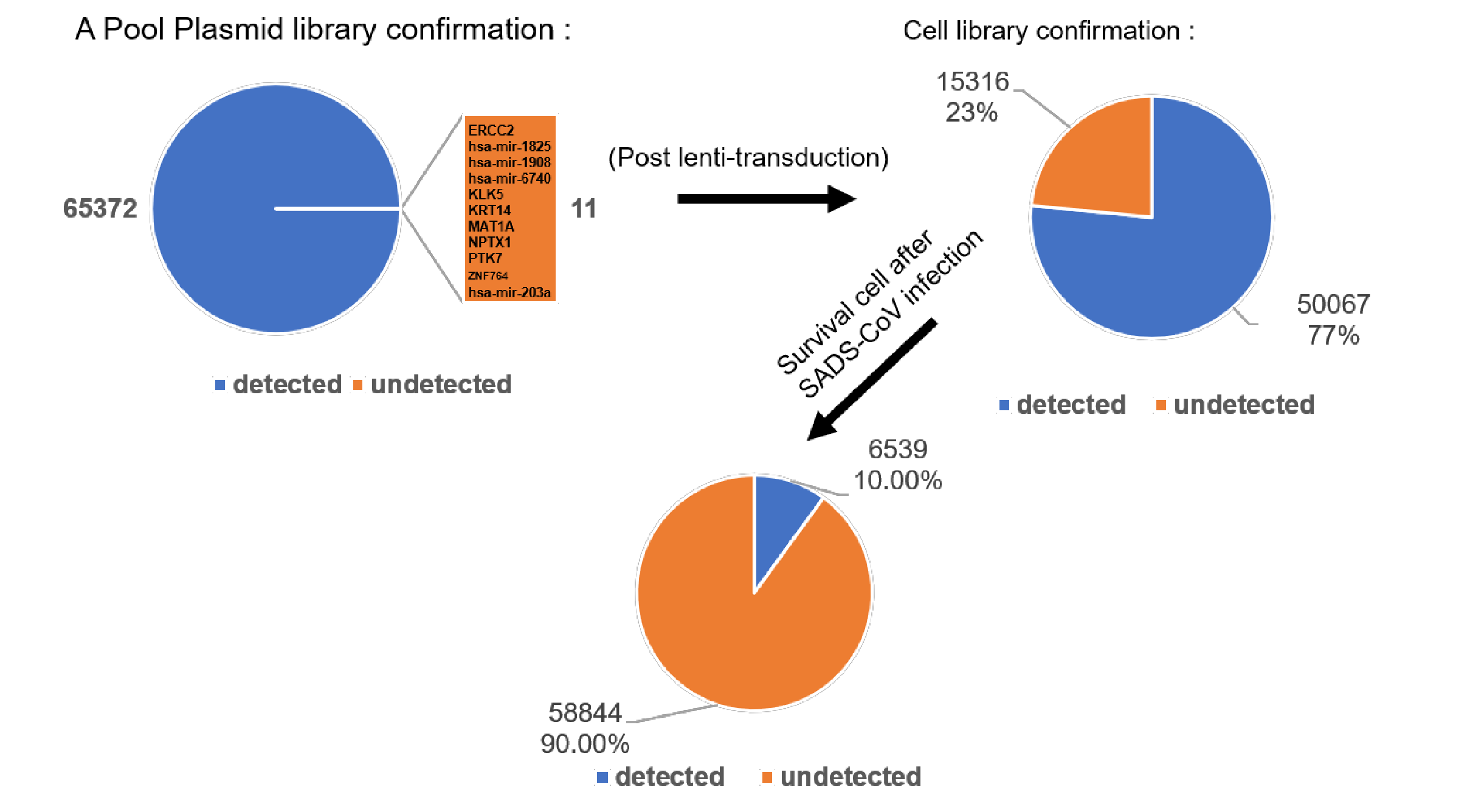

Supplement: FIG S1 [file mbio.02342-21-sf001.tif]

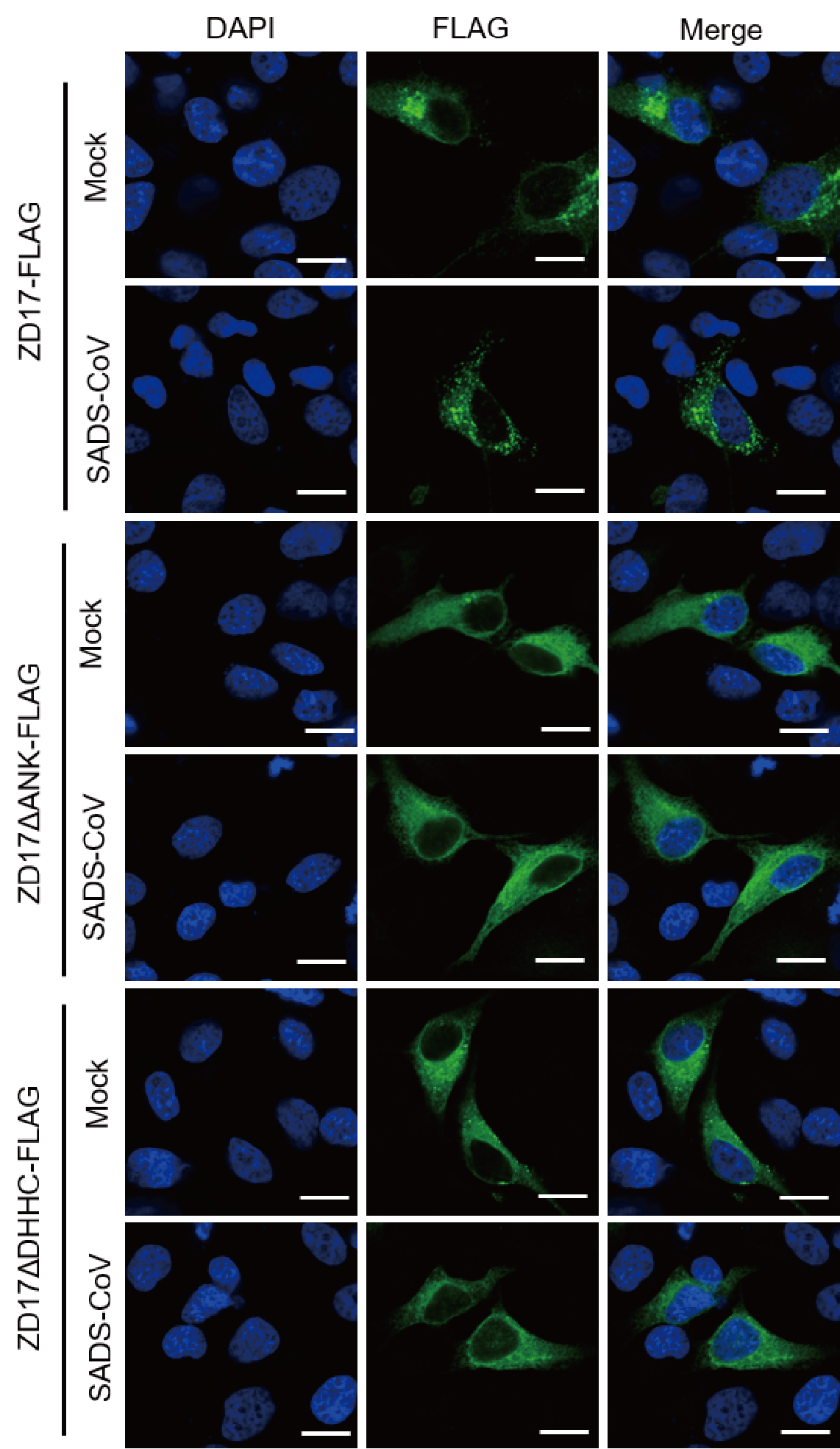

Supplement: FIG S2 [file mbio.02342-21-sf002.tif]

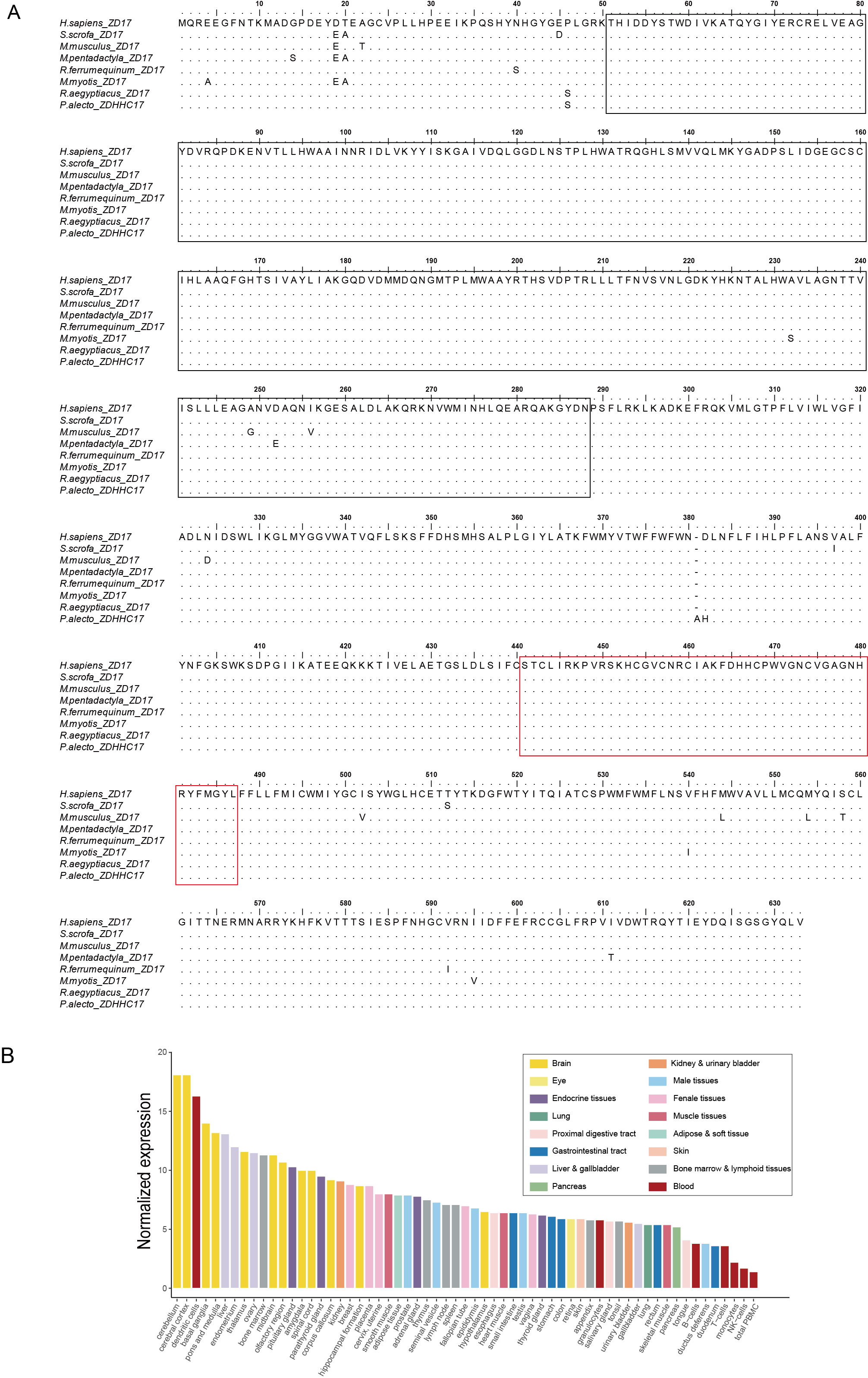

Supplement: FIG S3 [file mbio.02342-21-sf003.tif]
