# Supplementary material for: Identification of ZDHHC17 as a Potential Drug Target for Swine Acute Diarrhea Syndrome Coronavirus Infection
Source: mBio. 2021 Oct 26;12(5):e02342-21. doi: 10.1128/mBio.02342-21 (PMC8546599; doi:10.1128/mBio.02342-21)
Supplement: TABLE S1 [file mbio.02342-21-st001.docx]

**Supplementary table S1**. Palmitoylation sites of nsp3, nsp4 and nsp6.

| Protein | Position | Peptide | Score | Cutoff |
| --- | --- | --- | --- | --- |
| SADS-CoV_Nsp3 | 17 | ISDAVNFCKNLRLHF | 24.816 | 4.222 |
| SADS-CoV_Nsp3 | 1419 | DKPSCVACCKSAKLK | 9.898 | 3.419 |
| SADS-CoV_Nsp3 | 1420 | DKPSCVACCKSAKLK | 8.66 | 4.222 |
| SADS-CoV_Nsp4 | — | — | — | — |
| SADS-CoV_Nsp6 | — | — | — | — |

Protein palmitoylation sites of nsp3, nsp4 and nsp6 were predicted via (<http://csspalm.biocuckoo.org/>). The potential palmitoylation sites were highlighted by red.
